# Targeting NF-κB Signaling: Selected Small Molecules Downregulate Pro-Inflammatory Cytokines in Both Food Allergen and LPS-Induced Inflammation

**DOI:** 10.3390/ijms25115798

**Published:** 2024-05-26

**Authors:** Milena Zlatanova, Andrijana Nešić, Jovana Trbojević-Ivić, Danilo Četić, Marija Gavrović-Jankulović

**Affiliations:** 1 Department of Biochemistry, Faculty of Chemistry, University of Belgrade, 11000 Belgrade, Serbia; zlatanova@chem.bg.ac.rs (M.Z.); anesic@chem.bg.ac.rs (A.N.); 2 Institute for Translational Medicine (ITM), Medical School Hamburg (MSH), 20457 Hamburg, Germany; 3 Innovative Centre, Faculty of Chemistry, Belgrade, Ltd., 11000 Belgrade, Serbia; jivic@chem.bg.ac.rs; 4 Department for Metabolism, Institute for the Application of Nuclear Energy, University of Belgrade, 11000 Belgrade, Serbia; danilo.cetic@inep.co.rs

**Keywords:** NF-κB, immunomodulation, pro-inflammatory cytokines, allergic inflammation, vanillyl alcohol, lauric acid, nutraceuticals

## Abstract

Although inflammation is primarily a protective response guarding the human body, it can result in a variety of chronic diseases such as allergies, auto-immune, cardiovascular diseases, and cancer. In NF-κB-mediated inflammation, many small molecules and food compounds characterized as nutraceuticals have shown positive effects associated with immunomodulatory properties. We investigated the effects of selected bioactive small molecules, commonly found in food components, vanillyl alcohol (VA) and lauric acid (LA), on different cell lines exposed to pro-inflammatory stimuli, lipopolysaccharide (LPS), and the food allergen actinidin (Act d 1). Pro-inflammatory cytokines were downregulated in response to both VA and LA, and this downregulation was caused by a decrease in the activation of the NF-κB pathway and the translocation of p65, the pathway’s major component. Small nutraceutical molecules, VA and LA, showed not only inhibition of the pro-inflammatory cytokines, but also inhibition of the NF-κB activation, and reduced translocation of the p65 component. The present study may contribute to the therapeutic use of these molecules for various inflammatory diseases, which have in common an increased expression of pro-inflammatory cytokines and NF-κB-mediated inflammation.

## 1. Introduction

Inflammation is an essential survival mechanism that has evolved to eliminate tissue damage caused by pathogens and chemical or mechanical stress. Excessive production of pro-inflammatory cytokines, chemokines, and reactive oxygen species (ROS), which are primarily secreted by immune cells such as macrophages, can cause severe damage and contribute to the pathogenesis of a variety of inflammatory diseases [1,2]. As a result, anti-inflammatory agents are being extensively studied as potential therapeutic agents for the prevention and treatment of chronic inflammatory damage.

Nuclear factor-κB (NF-κB) is a key mediator of inflammatory responses and a regulator of innate and adaptive immune functions [3,4,5]. Five structurally related members include NF-ĸB1 (p50), NF-ĸB2 (p52), RelA (p65), RelB, and c-Rel, which mediate the transcription of target genes by binding to a specific DNA element in the nucleus. Inactive NF-ĸB components are localized in the cytoplasm, complexed with inhibitory proteins, such as IĸB family members [3,6]. Upon NF-ĸB activation, IĸB kinase is phosphorylated and NF-ĸB dimers (predominantly composed of p50 and p65) are subsequently translocated to the nucleus [3]. Overwhelming evidence supports the important role of NF-ĸB p65 in the pathogenesis of autoimmune diseases, allergies, and inflammatory bowel disease, commonly caused by the excessive production of pro-inflammatory cytokines and its activation by different proteins or lipids found in food components [3,7,8].

Food allergy is an inappropriate immune response to food proteins listed as allergens. In atopic individuals, food allergens trigger an immune response and induce allergic inflammation when they come in contact with professional antigen-presenting cells, but also at the site of the intestinal mucosa, where they interact with epithelial cells of the intestinal barrier and lead to the increased secretion of cytokines in the gut [9,10]. Actinidin (Act d 1) is a major kiwifruit allergen that belongs to the C1 cysteine protease family (EC 3.4.22.14) and retains its enzymatic activity even after the harsh conditions in the gastrointestinal tract, where it mediates the upregulation of the pro-allergenic cytokines, possibly via NF-κB activation. Its primary pro-inflammatory mechanism has been suggested to stem from its enzymatic activity. This notion is supported by the absence of inflammation and cytokine secretion when the enzyme is inhibited by a specific cysteine protease inhibitor [10,11,12].

Food allergens and other food components have been shown to directly permeate the intestinal barrier through intestinal epithelial cells. For this reason, it is crucial to investigate how food allergens affect epithelial cell lines such as human epithelial colorectal adenocarcinoma cells (Caco-2) and human embryonic kidney epithelial-like HEK293 cells [13,14]. However, potential immunomodulation and further interactions of the food allergens with immune cells have been less commonly studied. In vitro cell lines, such as human leukaemia monocytes (THP-1 cell line), are suitable and reliable models for studying monocyte and macrophage responses and have been proposed as a useful screening tool for potential immunomodulatory food compounds [15,16]. Also, the function and underlying mechanism of the NF-ĸB pathway have been extensively studied in macrophages, immune cells residing in tissues across the body, as a frontline immune response [10,12,17].

Many food compounds characterized as nutraceuticals and plant extracts have shown positive effects associated with immunomodulatory properties in a variety of in vitro and animal studies. Vanillyl alcohol (VA, 4-hydroxy-3-methoxybenzyl alcohol) and its oxidized forms, vanillin (VN) and vanillic acid (VAc) are phenolic active compounds commonly found in several medicinal plants (*Gastrodia elata*, *Vanilla planifolia*, etc.). Plant extracts containing VA and VN have proven anti-angiogenic, anti-inflammatory, and antioxidant properties [18,19,20,21,22,23]. Lauric acid (LA, dodecanoic acid) is a medium-chain saturated fatty acid with recognized antibacterial activity, as well as complex metabolic effects. It is commonly found in a wide variety of foods like coconut oil, fruits, seeds, and milk [24,25]. A limitation of the current studies is that most of them examine their anti-inflammatory effect induced by strong pro-inflammatory stimuli such as high concentrations of LPS (lipopolysaccharide) and/or focus on research in mice models [26,27,28]. Furthermore, the biological effects are usually studied in the context of complex medicinal plant extracts, which makes it challenging to discern the link between the specific molecule and the corresponding biological effect. In that sense, our study will provide the specific physiological mechanism to the concrete compound tested, while also using stimuli with a moderate inflammatory potential, such as food allergens.

In this study, we aimed to achieve the following: (a) develop a suitable model system for the analysis of pro-inflammatory stimuli on THP-1 macrophages; (b) to examine the immunomodulatory and inhibitory effects of VA and LA on pro-inflammatory response in THP-1 macrophages, HEK293 cells, and Caco-2 cells, and (c) to test the inhibitory potential of these molecules on Act d 1- and LPS-mediated NF-ĸB activation by analyzing the content and expression levels of the p65 component.

## 2. Results

### 2.1. Both LPS and Allergen Act d 1 Trigger Inflammation in THP-1 Differentiated Macrophages under Optimized Treatment Conditions

**Figure 1:**
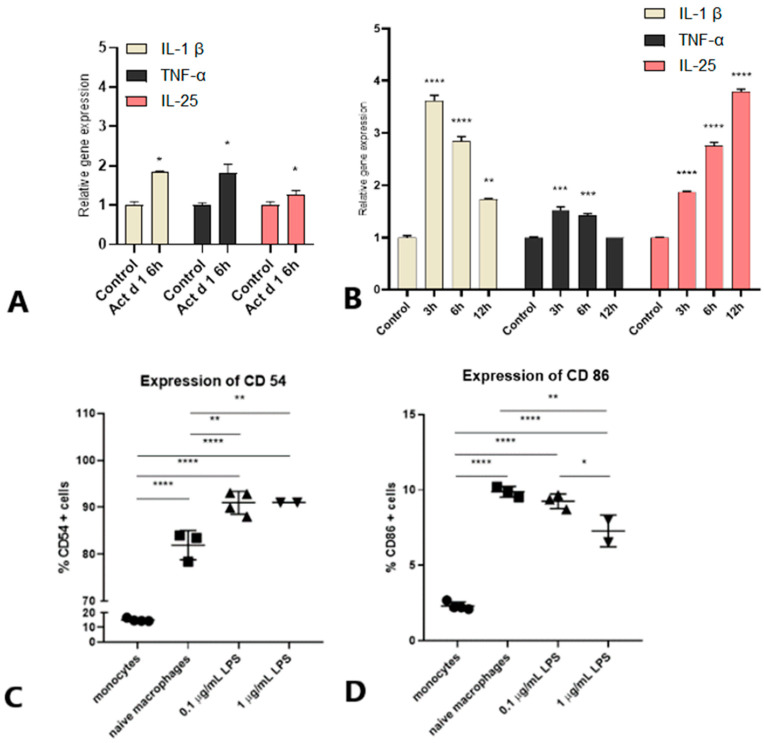
Treatment conditions were optimized to examine cytokine expression in THP-1 macrophages differentiated with 100 ng/mL PMA. (**A**) Act d 1 (16.62 µM) induced upregulation of IL-1β, TNF-α, and IL-25 after 6 h. (**B**) Kinetics of IL-1β, TNF-α, and IL-25 upregulation after treatment with 0.1 µg/mL LPS for 3, 6, and 12 h. LPS treatment induced the expression of cell surface markers in THP-1 macrophages. The expression of (**C**) CD54 and (**D**) CD86 was analysed in THP-1 monocytes, naïve untreated macrophages, and macrophages treated with 0.1 and 1 µg/mL LPS for 24 h. The treatment time was extended to allow the expression of surface markers, FITC-positive cells were gated on the FL1H channel, and results were presented as a percentage of CD54 or CD86 positive cells per total cell population. The results are presented as mean ± SEM; * *p* value < 0.1, ** *p* value < 0.01, *** *p* value < 0.001, and **** *p* value < 0.0001.

THP-1 monocytes were differentiated into macrophages with 100 ng/mL PMA. Our previous study showed that after 6 h of treatment, 16.62 µM Act d 1 efficiently upregulates the expression of tight junction (TJ) proteins and cytokines in epithelial cells [29]. We wanted to test if the same treatment would affect cytokine expression in PMA-differentiated THP-1 macrophages. Indeed, after 6 h of treatment with 16.62 µM Act d 1, we observed a significant increase in all analyzed pro-inflammatory cytokines (Figure 1A). 

Further, we investigated whether and at what time points a concentration of 0.1 µg/mL LPS could cause detectable changes in the cytokine gene expression. This concentration was found to be sufficient to induce changes in THP-1 macrophages differentiated with 100 ng/mL PMA (Figure 1B). The expression kinetics of different cytokines vary, but our findings show that levels of IL-1β and TNF-α peak at 3 h, then decrease slightly after 6 h, and further decrease after 12 h of LPS treatment. In terms of IL-25, the expression increased steadily over time. These findings are consistent with previous research, which found that IL-1β and TNF-α peak at around 4 h of treatment [30]. In summary, after 6 h of LPS treatment all cytokines were upregulated. 

The phenotypic differences between monocytes and differentiated macrophages, as well as their response to lower concentrations of LPS, were analyzed by the expression of two cell surface markers, CD54, the intercellular adhesion molecule-1 (ICAM-1), and CD86, the costimulatory molecule B7-2. The percentage of CD54 and CD86 positive cells increased significantly after differentiation (Figure 1C,D), which highlights the difference of surface receptors in THP-1 monocytes compared to THP-1 macrophages. LPS at both concentrations increased the levels of CD54 surface molecules (Figure 1C). On the other hand, higher LPS concentrations resulted in a modest decrease in CD86 levels when compared to naïve untreated macrophages (Figure 1D).

### 2.2. VA and LA Have No Impact on Cell Viability or Pro-Inflammatory Effect

**Figure 2:**
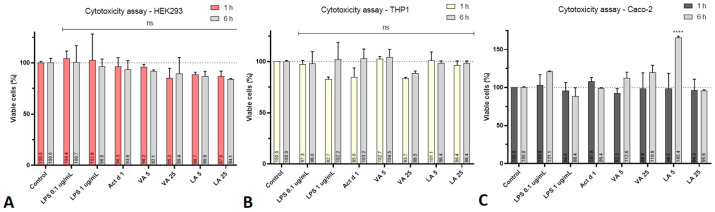
Effect of Act d 1, LPS, VA, and LA on the viability of (**A**) HEK293 cells, (**B**) THP-1 macrophages, and (**C**) Caco-2 cells after 1 h and 6 h of treatment. Results are presented as mean ± SEM; ns—non significant, **** *p* value < 0.0001, compared to control.

The CCK-8 cytotoxicity assay was employed to assess the cytotoxicity of all molecules used in cell treatments. LPS (0.1 and 1 µg/mL), Act d 1 (16.62 µM), VA (5 and 25 µM), and LA (5 and 25 µM) were applied to the cells for 1 and 6 h, respectively. During this time, none of the examined compounds demonstrated statistically significant cytotoxicity to HEK293 cells, Caco-2 cells, or THP-1-differentiated macrophages at the tested concentrations (Figure 2). The only statistically significant changes were noticed in Caco-2 cells, where there was a positive effect on cell proliferation after 6 h of treatment with the lower concentration of lauric acid (Figure 2C). 

**Figure 3:**
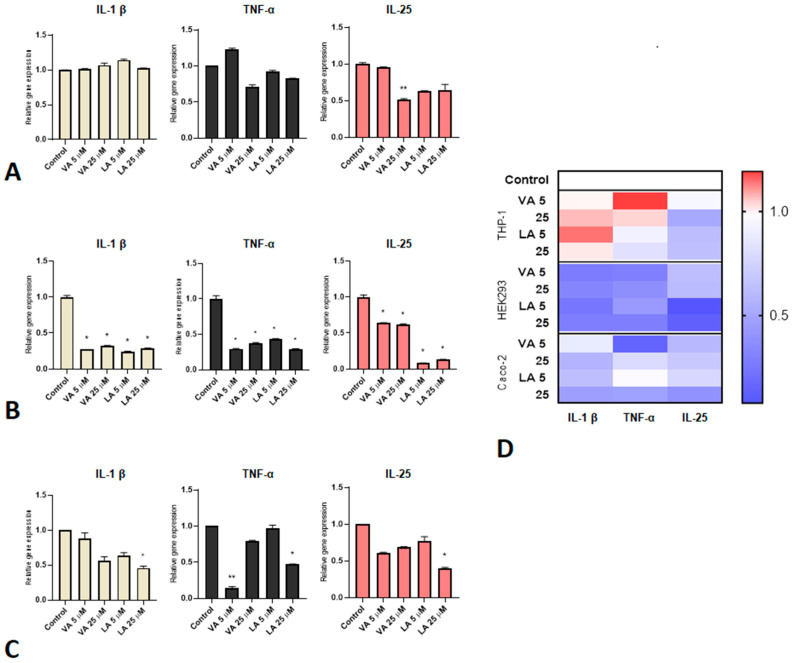
Effect of VA and LA on the expression of genes that promote inflammation. The effect of different concentrations of VA and LA on the expression of IL-1β, TNF-α, and IL-25 in (**A**) THP-1 macrophages, (**B**) HEK293 cells, and (**C**) Caco-2 cells. A heat map (**D**) of the inflammatory response based on the cytokine gene expression in all cell lines is also presented. The cells were treated for 6 h with different concentrations of VA and LA. The results are presented as mean ± SEM; * *p* value < 0.1 and ** *p* value < 0.01, compared to control.

The gene expression of the pro-inflammatory cytokines IL-1β, TNF-α, and IL-25 was measured after the treatment of THP-1, HEK293, and Caco-2 cells with VA and LA in two different concentrations (5 and 25 µM) to see if these molecules have pro-inflammatory or anti-inflammatory effects. Cytokine expression was not upregulated. The downregulation in THP-1 cells was not statistically significant, except IL-25 expression after treatment with the higher concentration of VA, but the cytokine expression overall remained unchanged after VA and LA treatment compared to control (Figure 3A). In Caco-2 cells, there was a drop in TNF-α expression after the treatment with 5 µM VA, as well as with the higher concentration of LA (25 µM), which led to a significant decrease in the expression of all three analyzed cytokines (Figure 3C). Meanwhile, in HEK293 cells, all cytokines were downregulated with both VA and LA, indicating their anti-inflammatory potential (Figure 3B).

### 2.3. Small Molecules Reduce the Expression of Pro-Inflammatory Cytokines in Inflammation Responses

**Figure 4:**
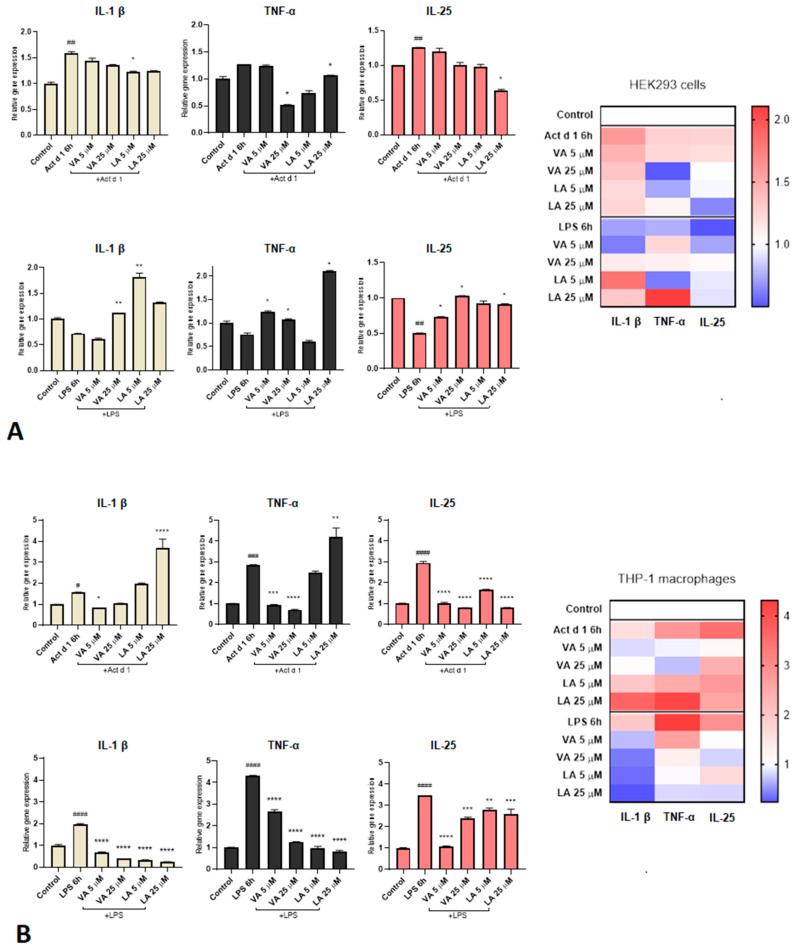

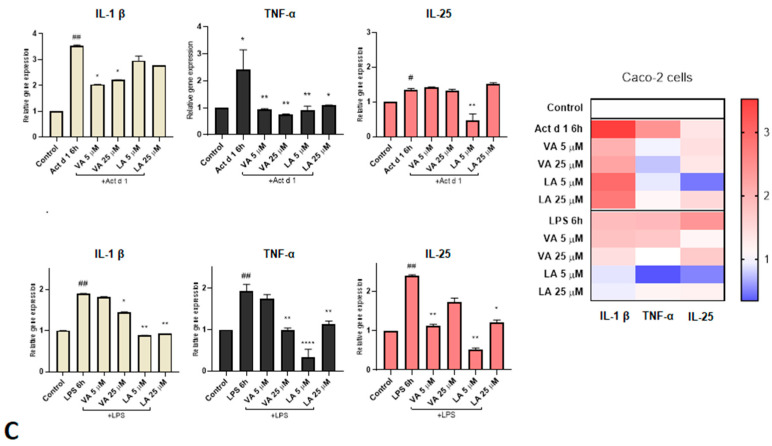
Modulation of the expression of pro-inflammatory cytokine genes (IL-1β, TNF-α, and IL-25). HEK293 cells (**A**), THP-1 cells (**B**), and Caco-2 cells (**C**), were pretreated with different concentrations of VA and LA and then stimulated with 16.62 μM Act d 1 and 0.1 μg/mL LPS. A heat map of cytokine expressions is also shown for all three cell lines. The results are compared to LPS/Act d 1 * or compared to control #. The results are presented as mean ± SEM; */# *p* value < 0.1, **/## *p* value < 0.01, ***/### *p* value < 0.001, and ****/#### *p* value < 0.0001.

In order to examine the effect of VA and LA in an immune response to pro-inflammatory stimuli, cells were treated with 0.1 µg/mL LPS or 16.62 µM Act d 1 without or after pretreatment with two different concentrations of small molecules (5 and 25 µM) (Figure 4). Following the stimulation with both Act d 1 and LPS, the THP-1-derived macrophages showed an increased expression of all three analyzed cytokine genes (IL-1β, TNF-α, and IL-25). Both concentrations of VA pretreatment reduced Act d 1-induced IL-1β, TNF-α, and IL-25 gene expression. However, LA did not reduce IL-1β and TNF-α expression but did reduce IL-25 gene expression (Figure 4B). LPS-induced expression of IL-1β, TNF-α, and IL-25, on the other hand, was downregulated by both VA and LA (Figure 4B). In HEK293 cells, only Act d 1 increased the expression of IL-1β and IL-25, but not TNF-α (Figure 4A). In line with the literature’s data, there was no statistically significant increase in TNF-α production in HEK293 cells, which was addressed in TNF-α studies by using stable transfected TNF-α-HEK293 clones [31]. Small molecules’ inhibitory potential was less pronounced in this cell model than in THP-1 macrophages, and it was only noticeable at a higher dosage (25 µM VA decreased levels of TNF-α, while 25 µM LA decreased levels of TNF-α and IL-25) (Figure 4A). In HEK293 cells after LPS treatment, none of the analyzed cytokines showed increased expression (Figure 4A). The lack of response to LPS in the HEK293 cells is presumably due to very low expression of TLR4 receptors on these cells [32]. In Caco-2 cells (Figure 4C), after Act d 1 treatment there was a decrease in IL-1β and TNF-α with both concentrations of VA, while in the LPS group there was a significant decrease in these cytokines with the higher concentration of VA (25 µM). On the other hand, LA led to a decrease in TNF-α in both concentrations after LPS and Act d 1 treatment, while IL-1β was decreased only after LPS treatment, which is consistent with the results from the THP-1 cell line. IL-25 was decreased with 5 µM LA in both Act d 1 and LPS treatments. 

### 2.4. Small Molecules Attenuate NF-ĸB Activation in HEK293 Cells

**Figure 5:**
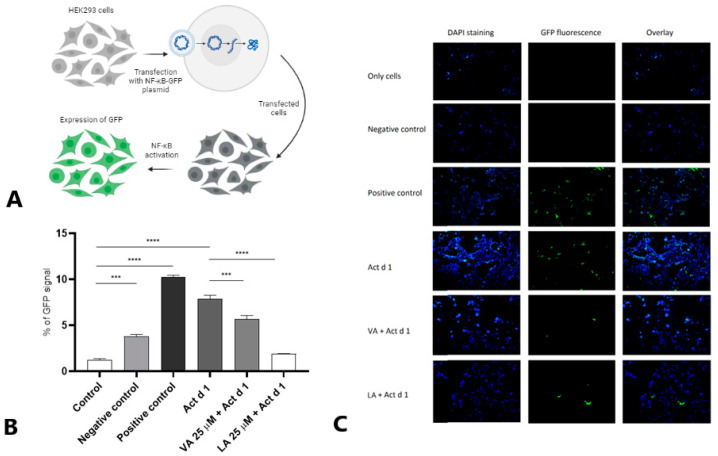
Levels of NF-κB activation in HEK293 cells. HEK293 cells were transfected with NF-κB-GFP plasmid (**A**) (created with Biorender.com) and treated with Act d 1 with or without pretreatment with VA and LA. Flow cytometry (**B**) was used to measure the GFP expression. Fluorescence microscopy (**C**) was used to visualize GFP expression in HEK293 cells after transfection with the NF-κB-GFP plasmid. Images were acquired by an objective with 60× magnification. The results are presented as mean ± SEM; *** *p* value < 0.001 and **** *p* value < 0.0001.

To analyze the ability of VA and LA to modulate the NF-κB activation in HEK293 cells, they were transfected with NF-κB-GFP plasmid (Figure 5A) and subsequently treated with 16.62 μM Act d 1 with or without pretreatment with 25 μM of VA or LA. Flow cytometry (Figure 5B) and fluorescence microscopy (Figure 5C) showed that higher concentrations of VA and LA inhibited Act d 1-mediated NF-ĸB activation in HEK293 cells pre-transfected with the NF-ĸB-GFP plasmid. In this experiment, LA had a more pronounced inhibitory effect, reducing NF-ĸB activation to the same extent as the negative control, whereas VA decreased NF-ĸB activation when compared to Act d 1.

### 2.5. Small Molecules Reduce the NF-ĸB p65 Levels in THP-1-Derived Macrophages

**Figure 6:**
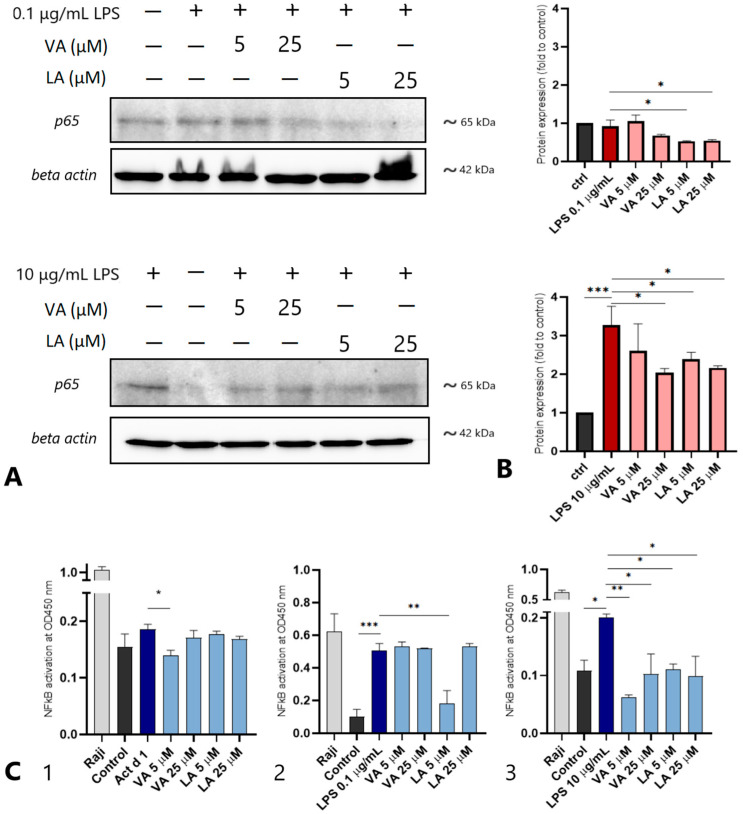
VA and LA modulate expression and translocation of the p65 component. The protein levels of p65 and β-actin were analyzed by (**A**) Western blot in THP-1 macrophages pretreated with VA or LA for 1 h and then treated with LPS (0.1 or 10 μg/mL); (**B**) a densitometric analysis was used to quantify the protein levels of p65. After treatment with pro-inflammatory stimuli, VA, and LA, the amount of p65 was measured in nuclear extracts using the NF-κB transcription factor assay kit (**C**). Cells were pretreated with 5 or 25 μM of small molecules and subsequently treated with 16.62 μM Act d 1 (**C1**), 0.1 µg/mL (**C2**), and 10 μg/mL LPS (**C3**) as pro-inflammatory stimuli. The results are presented as mean ± SEM; * *p* value < 0.1, ** *p* value < 0.01, and *** *p* value < 0.001.

To further investigate the inhibitory effects of VA and LA, we used Western blot to examine the expression of p65 in the total cell lysates. For NF-κB activation, the Act d 1 concentration (16.62 μM) remained unchanged from previous experiments, as did the LPS concentration (0.1 μg/mL). Furthermore, we used a higher LPS concentration (10 μg/mL) that had previously been used in similar experiments reported by Saha et al. [33]. Act d 1 treatment did not produce bands of sufficient intensity, and thus could not be analyzed using the Western blot method. However, both LPS concentrations resulted in clear bands with a molecular weight of 65 kDa (Figure 6A), and a densitometric analysis was used to quantify p65 expression (Figure 6B). The expression of β-actin was analyzed for normalization of the p65 expression. In the LPS-treated group, p65 expression increased after the treatment with a higher concentration of LPS (10 μg/mL), and the expression statistically decreased after pretreatment with both VA (25 μM) and LA (5 and 25 μM). We found no statistical difference in the p65 expression between the control and the cells treated with a lower concentration of LPS (0.1 µg/mL).

In order to analyze the effect of VA and LA on the translocation of the activated p65 into the nuclei, the amount of p65 in the nuclear lysates alone was determined by ELISA with coupled NF-κB-specific oligonucleotide sequences. When compared to the untreated cell group, Act d 1 treatment resulted in a slight increase in nuclear p65. However, after treatment with 5 μM VA, nuclear p65 levels were significantly lower than those in the Act d 1 group (Figure 6C1). Nuclear p65 increased in the LPS-treated group at both concentrations. Only after pretreatment with 5 µM LA (Figure 6C2), a significant decrease in nuclear p65 in the cells treated with lower concentrations of LPS (0.1 µg/mL) was observed. When the higher LPS concentration of 10 µg/mL was used for stimulation, both VA and LA were found to negatively regulate nuclear p65 and have an inhibitory effect on the NF-ĸB activation, as nuclear p65 was reduced upon treatment with VA and LA at both concentrations tested (Figure 6C3).

## 3. Discussion

We have designed a sensitive in vitro model system to examine the modulation of different pro-inflammatory stimuli such as LPS and food allergens that act as moderate pro-inflammatory stimuli, on macrophages differentiated from THP-1 monocytes. A sensitive macrophage model system with increased expression of the pro-inflammatory cytokine genes IL-1β, TNF-α, and IL-25 was obtained using a lower concentration of LPS and food protease Act d 1. We also looked at phenotypic changes in the surface markers CD54 and CD86 and found that both were upregulated in the THP-1 macrophages compared to the undifferentiated monocytes. These findings clearly support the plasticity of the THP-1-derived macrophages and their ability to differentiate further in response to various stimuli, altering their phenotype and cytokine expression [34,35]. The purified and activated food protease and allergen Act d 1 induced gene expression of the analyzed cytokines IL-1β, TNF-α, and IL-25 in the treated THP-1 macrophages. This study confirms previous findings on epithelial cells and emphasizes the pro-inflammatory effect of active Act d 1, as well as its role in NF-ĸB activation [10], but also further characterizes the NF-ĸB activation as occurring through the classical NF-ĸB pathway, which has p65 as its main component. These findings further emphasize the pro-inflammatory effect of food proteases, commonly major food allergens, and the potential damage that can be caused, especially in sensitized individuals. 

The anti-inflammatory effects of different nutrients and bioactive small molecules from plant extracts on the LPS-induced inflammation have been the subject of numerous studies [17,36,37]. Furthermore, few studies on non-LPS stimuli such as allergens and the immunomodulatory potential of selected small bioactive molecules in allergic inflammation have been conducted [38]. In our study, the expression of the three cytokines analyzed in THP-1 cells was unchanged compared to control. The same trend was noticed in Caco-2 cells, except the downregulation of all cytokines with the higher concentration of LA, whereas in HEK293 cells, all cytokines were downregulated with both VA and LA, indicating their anti-inflammatory potential. Furthermore, we discovered that VA and LA reduced the expression of pro-inflammatory genes such as IL-1β, TNF-α, and IL-25 in THP-1 macrophages during the LPS-induced inflammation. The downregulation of the LPS-induced inflammation was noticed in Caco-2 cells as well, where LA had a more pronounced effect in reducing the levels of all three cytokines in both concentrations. The results for VA presented in this study are in excellent agreement with the previous research elucidating the anti-inflammatory effects and mechanism of VN and VAc, the oxidized forms of VA [21,22,39]. The results observed for the effect of LA are consistent with the previously reported LA-mediated downregulation of IL-6 and IL-8 in THP-1 cells during induced skin inflammation [40]. In contrast, we found only downregulation of IL-1β and TNF-α after pretreatment with VA, but not with LA, in Act d 1-mediated inflammation in THP-1 macrophages. The same trend could be observed in Caco-2 cells’ expression of IL-1β. In Act d 1-mediated inflammation in HEK293 cells, LA reduced the expression of all the cytokines tested, whereas VA statistically reduced the expression of only TNF-α. The lack of response to LPS in the HEK293 cells is presumably due to the very low expression of TLR4 receptors on these cells [32]. The slight difference in the response to the tested molecules in the different cell lines can be explained in a similar manner. One of the suggested mechanisms is that vanilloids and their counterparts bind to transient receptor potential vanilloid 1 (TRPV1) [41]. TRPV1 is primarily expressed in sensory neurons, but it has also been found in non-neuronal cells and immune cells such as dendritic cells, macrophages, and T-cells [41,42]. The difference in TRPV1 expression levels between macrophages and epithelial cells could be one of the reasons why we observe a slightly different immunomodulatory potential of these small molecules in different cell lines. The findings for LA in Act d 1-induced inflammation in HEK293 cells are consistent with the findings of a recent in vivo study in mice models, which revealed that zinc-laurate exerts anti-inflammatory activity via downregulation of IL-1β and TNF-α [43]. As a result, our findings provide an excellent foundation for further investigation of LA nutraceutical potential in the treatment of chronic inflammation of the gastrointestinal tract. 

Although we have shown that these two small molecules have inhibitory potential in all three cell lines, the effect in HEK293 cells is less pronounced, which could be due to the difference in surface receptor expression. The NF-ĸB inhibitory potential of VA and LA in HEK293 cells was demonstrated using the NF-ĸB-GFP plasmid. In comparison to Act d 1-treated cells, which showed increased NF-ĸB activation, cells pretreated with each of the small molecules showed decreased activation. However, nuclear localization of p65 in THP-1 macrophages was reduced more significantly after treatment with VA. These data are consistent with the expression levels of IL-1β and TNF-α, which were not reduced in THP-1 cells after pretreatment with LA. These findings are in agreement with the previous research suggesting that some food allergens have the ability to bind small molecules such as fatty acids, which contributes to their stability and changes in their conformation that can influence the sensitization process and binding of the molecules on different surface receptors [44]. This could explain why we observe the upregulation of IL-1β and TNF-α in THP-1 cells at the higher LA concentration only after Act d 1 treatment. The amount of nuclear p65 in THP-1 macrophages increased with the higher LPS concentration, and decreased in the cells pretreated with VA and LA at all concentrations tested, whereas the increased expression of p65 in total lysates was observed only after treatment with the increasing LPS concentration, and was downregulated with both VA and LA.

**Figure 7:**
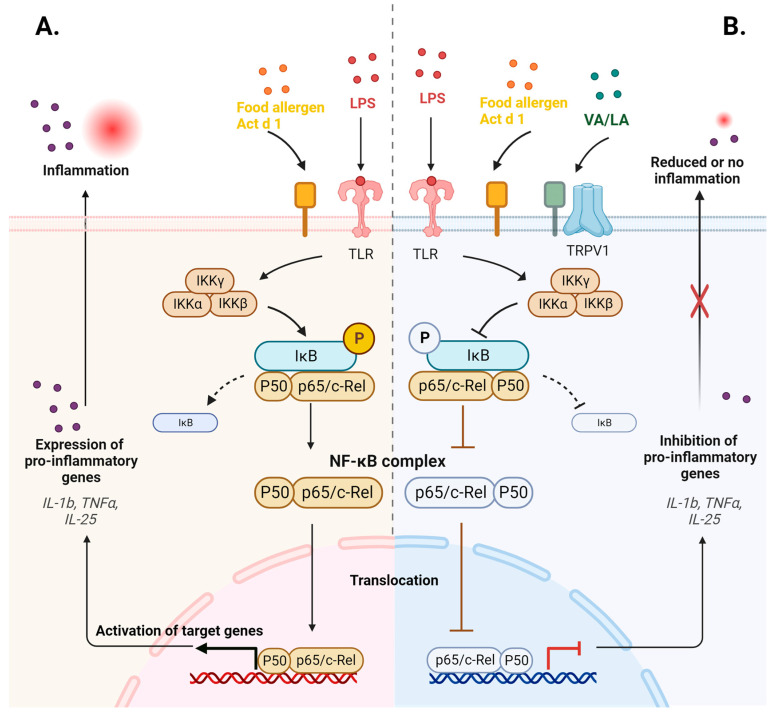
Inflammation induced by LPS and food allergen Act d 1 (**A**) is inhibited by VA and LA (**B**) via the downregulation of pro-inflammatory genes and NF-κB inhibition, mainly by inhibiting p65 activation and translocation (blunt arrows were used to illustrate the inhibitory effect). Created and adapted from template “Suppression of Inflammasome by IRF4 and IRF8 is Critical for T Cell Priming”, by Biorender.com. Retrieved from https://app.biorender.com/biorender-templates, accessed on 3 April 2024.

Overall, the search for effective small-molecule NF-κB inhibitors is ongoing, and additional research would confirm the anti-inflammatory effects of VA and LA in pathological conditions other than allergen and LPS-induced inflammation (Figure 7). 

In conclusion, our research strongly supports the idea and significant potential of plant and food-derived small molecules, such as VA and LA, in exerting beneficial anti-inflammatory effects across various cell types. These molecules demonstrate the ability to successfully modulate immune responses characterized by the elevated expression of pro-inflammatory cytokines, which acts as the base for many pathological conditions. We also highlight the role of the classical NF-κB signaling pathway and its subunit p65 due to its strong correlation with many inflammatory conditions and diseases [45], as well as the need for and potential benefit of an NF-κB inhibitor. To our knowledge, this is the first study investigating the immunomodulatory effects of small molecules and nutraceuticals like VA and LA on NF-κB signaling in a food allergen and food protease model. We believe that the results presented here, as well as the derived putative mechanism of VA and LA anti-inflammatory activity, provide a solid foundation for further in-depth molecular studies of many other potential small-molecule NF-ĸB inhibitors as therapeutic or nutraceutical agents for various inflammatory diseases, especially anti-allergic properties in inflammation characterized by elevated expression of pro-inflammatory cytokines.

## 4. Materials and Methods

### 4.1. Act d 1 Purification and Small Molecules’ Preparation

Act d 1 was isolated and purified from fresh kiwifruit (*Actinidia deliciosa,* Hayward cv) using two consecutive ion-exchange chromatographies, as previously reported [13,46]. A total of 75 mg of Act d 1 was obtained from 250 g of kiwifruit. The purified allergen was pooled and concentrated to a final concentration of 1 mg/mL, as determined by the Bradford assay, and lyophilized and stored at −20 °C. Protein purity was estimated at >95% by SDS-PAGE. The proteolytic activity of Act d 1 was assessed using an enzymatic assay with casein as a substrate [47].

Vanillyl alcohol (≥98% purity, food grade) and lauric acid (≥98% purity, food grade) were purchased from Sigma-Aldrich, St. Louis, MO, USA. For treatment purposes, they were dissolved in 0.05% DMSO in phosphate-buffered saline pH 7.4 to final concentrations of 5 and 25 µM. These concentrations were chosen as they appear in the literature as minimal concentrations for structurally analogous molecules that show physiological effects, but do not exhibit cytotoxic activity [37,38]. When we tested the concentrations of these molecules above 25 µM, we observed a decrease in cell viability.

### 4.2. THP-1 Monocytes and Their Differentiation into Macrophages

THP-1 human leukaemia monocytes (ATCC, Manassas, VA, USA) were grown and maintained in RPMI 1640 (Gibco, Thermo Fisher Scientific, Waltham, MA, USA), supplemented with 10% heat-inactivated fetal bovine serum (FBS, Biowest, Nuaille, France), penicillin (100IU)/streptomycin (100 µg/mL), 0.05 mM 2-mercaptoethanol, and 200 mM L-glutamine (Sigma-Aldrich, St. Louis, MO, USA). THP-1 cells are male. Cells were cultured in an atmosphere of 5% CO_2_ at 37 °C. THP-1 monocytes were differentiated into macrophages in 24-well plates at a cell density of 1 × 10^6^ cells/well in RPMI 1640 medium containing 100 ng/mL phorbol-12-myristate-13-acetate (PMA, Sigma-Aldrich, St. Louis, MO, USA) over 48 h. Thereafter, the cells were left to rest over 72 h in a complete RPMI 1640 medium.

### 4.3. HEK293 and Caco-2 Cell Culture

Human embryonic kidney epithelial-like HEK293 cells (ATCC, Manassas, VA, USA) were grown and maintained in Dulbecco’s modified Eagle’s medium (DMEM, Sigma-Aldrich, St. Louis, MO, USA), supplemented with 10% heat-inactivated fetal bovine serum (FBS, Biowest, Nuaille, France), penicillin (100IU)/streptomycin (100 µg/mL), and 200 mM L-glutamine (Sigma-Aldrich, St. Louis, MO, USA) in an atmosphere of 5% CO_2_ at 37 °C. Cells were passaged using 0.25% trypsin/0.53 mM EDTA solution (Sigma-Aldrich, St. Louis, MO, USA). HEK293 are female fetal cells.

Human epithelial colorectal adenocarcinoma (Caco-2) cells (ATCC, Manassas, VA, USA) were cultured in Eagle’s Minimum Essential Medium (EMEM, Sigma-Aldrich, St. Louis, MO, USA), supplemented with 20% heat-inactivated fetal bovine serum (FBS, Biowest, Nuaille, France), penicillin (100IU)/streptomycin (100 µg/mL), and 200 mM L-glutamine (Sigma-Aldrich, St. Louis, MO, USA) in an atmosphere of 5% CO_2_ at 37 °C. Cells were passaged using 0.25% trypsin/0.53 mM EDTA solution (Sigma-Aldrich, St. Louis, MO, USA). Caco-2 cells are male cells.

### 4.4. Cell Treatment

One hour before treatment, the medium was removed, and the cells were incubated in a serum-free medium for Act d 1 and a complete medium for LPS treatment. For the 96-well plate treatment, cells were seeded at a cell density of 5 × 10^3^ cells/well. For treatments in 24-well plates, cells were seeded at a cell density of 5 × 10^4^ cells/well, and cultivation was extended until confluency was reached. Prior to cell treatment, food allergen Act d 1 was activated in an appropriate cell culture medium containing cysteine for 1 h at 37 °C. The cell treatment concentration of 16.62 μM (1 mg/mL) Act d 1 was previously optimized on epithelial cell lines, as was the optimal treatment time of 6 h. LPS concentrations of 0.1 and 1 µg/mL are common concentrations used in other studies for cytokine expression analysis [48]. In all treatments, small molecule treatment began 1 h before LPS/Act d 1 treatment at two different concentrations, 5 and 25 μM, respectively. Act d 1 or LPS was added after 1 h, and the treatment was continued for 6 h for Act d 1 and 3, 6, or 12 h for LPS. Following this time period, the supernatants were removed and the cells were used. The time points of 3, 6, and 12 h were chosen because they were the most commonly used in studies investigating the kinetics of cytokine expression after LPS treatment [48,49]. Most studies range from 45 min to 2 h for pretreatment with potential inhibitory or anti-inflammatory small molecules, with 1 h being the most commonly used [37,38].

### 4.5. Flow Cytometric Analysis of THP-1-Derived Macrophages

The cell size, complexity, and expression of cell surface markers were measured by flow cytometry. THP-1 monocytes were cultured in T-75 flasks in a complete medium, and THP-derived macrophages were differentiated from monocytes and treated with 0.1 and 1 µg/mL LPS. Treatment time was extended to 24 h to allow the expression of surface markers. Cells were detached using Accutase (Biowest, Nuaille, France), and stained with anti-human CD54 (5 µL of commercial antibody per million cells) and anti-human CD86 FITC-labelled antibodies (5 µL of commercial antibody per million cells) (BioLegend, San Diego, CA, USA), respectively, for 20 min at 4 °C, according to the manufacturer’s recommendation. Cells were analyzed by flow cytometry using FACS Calibur (BD Biosciences, San Jose, CA, USA) equipped with a blue solid-state 200-mW laser (488 nm used for excitation) and an appropriate detection filter (525 nm, FL1). Raw data were analyzed using BD CellQuestPro software version 5.1 (BD Biosciences, San Jose, CA, USA).

### 4.6. Isolation of RNA and Gene Expression Analysis

Following treatment, the cell medium was collected, and the total RNA was isolated from THP-1 macrophages, HEK293, and Caco-2 epithelial cells using TRI reagent (Sigma-Aldrich, St. Louis, MO, USA). The total RNA (1.5 µg) extracted was used for reverse transcription employing random primers and the RevertAid First Strand cDNA Synthesis Kit (Thermo Fisher Scientific, Waltham, MA, USA). Following reverse transcription, the obtained cDNA was analyzed by PCR. The reaction mixture (20 µL total volume), prepared according to the manufacturer’s instructions, consisted of 500 ng of cDNA mixed with PCR MasterMix (Thermo Fisher Scientific, Waltham, MA, USA), forward and reverse primers (final concentration 300 nM per primer), and water. The parameters of the PCR cycle were as follows: initial denaturation at 95 °C for 5 min (1 cycle), followed by 35 cycles of denaturation at 95 °C for 60 s, annealing temperature (AT) for 60 s, and elongation at 72 °C for 30 s. Gene-specific primers and annealing temperatures for the human genes are listed in Table 1.

Following the completion of the PCR reaction, the presence, size, and quantity of the amplified PCR products were examined on 2% agarose/SimplySafe™ dye gels (EURx, Gdansk, Poland). The O’Gene Ruler 100 bp DNA Ladder (Fermentas, Vilnius, Lithuania) was used to confirm the molecular size of the PCR products. Two independent experiments were used for the quantification of the PCR bands using Image Studio Lite version 5.2 (LICOR, Biosciences, NE, USA). GAPDH was used as a housekeeping gene to allow the normalization of mRNA levels between samples, and mRNA levels measured upon treatment were compared with the untreated samples set to a value of 1.

### 4.7. CCK-8 Cytotoxicity Assay

To assess the viability of THP-1 macrophages, HEK293 and Caco-2 epithelial cells after treatment with VA, LA, Act d 1, and LPS, the Cell Counting Kit-8 was used (Sigma-Aldrich, St. Louis, MO, USA), as already described [46]. Cells were seeded in a 96-well plate at a cell density of 5 × 10^3^ cells/well. Monocytes were differentiated into macrophages as described previously, and HEK293 and Caco-2 cells were grown until they were attached to the well surface (24 h), as per the manufacturers’ protocol instruction. The cells were then exposed for 1 h and 6 h to one of the following: LA (5 µM and 25 µM), VA (5 µM and 25 µM), Act d 1 (16.62 µM), and LPS (0.1 µg/mL and 1 µg/mL), respectively. Following the specified time, 10 µL of CCK-8 was added to the wells and incubated for 4 h at 37 °C. A microplate reader (BioTek Synergy LX Multimode Reader, Agilent, CA, USA) was used to measure absorbance at 450 nm.

### 4.8. NF-κB-GFP Reporter Assay

As previously described, HEK293 epithelial cells were cultured for transient transfection and seeded in a complete growth medium in a 24-well plate with or without glass coverslips at a cell density of 3 × 10^4^ cells/well. The cells were grown until they reached ~80–90% confluence. A mixture of 0.4 µg/well of the appropriate DNA (Cignal NF-κB-GFP Pathway Reporter Assay Kit, Qiagen, Hilden, Germany) was mixed with 1.5 µL/well Attractene Transfection Reagent (Qiagen, Hilden, Germany), and incubated for 20 min at RT. Then, the transfection mixture was added into the appropriate wells, and the cells were incubated for 20 h. After 18 h of incubation, the medium was replaced with a fresh medium containing antibiotics. After 24 h of incubation, the cells were treated with activated Act d 1 (16.62 µM) for 24 h or with VA and LA for 1 h before treatment with activated Act d 1. Activation of the NF-κB-GFP was assessed by flow cytometry and by fluorescence microscopy.

Visualization of the NF-κB-GFP activation in transfected HEK293 cells was accomplished by fluorescence microscopy. The cells were first fixed on the coverslips using 4% paraformaldehyde (Fischer Scientific, Waltham, MA, USA). After staining the nuclei with DAPI reagent, the cells were analyzed using a fluorescence microscope (Opto-Edu, Beijing, China).

The fluorescence intensity of GFP was also measured using a FACS Calibur (BD Biosciences, San Jose, CA, USA) equipped with a blue solid-state 200-mW laser (488 nm used for excitation) and an appropriate detection filter (525 nm, FL1). BD CellQuestPro software version 5.1 (BD Biosciences, San Jose, CA, USA) was used to analyze the raw data.

### 4.9. Western Blot

To analyze the expression of p65 in THP-1 cells, Western blot was performed using a standard protocol. After treatment, cells were collected with Accutase and the cell pellet was resuspended in cold lysis buffer (20 mM Tris-HCl, 150 mM NaCl, 1% NP-40 with added protease and phosphatase inhibitors (Roche, Basel, Switzerland)). The BCA Protein Assay Kit (Thermo Fisher Scientific, Waltham, MA, USA) was used to determine the total protein concentration. Proteins were separated by 12% SDS-PAGE and transferred to the NC membrane. The membrane was blocked overnight with 2% BSA (Thermo Fisher Scientific, Waltham, MA, USA) and then incubated for 2 h at RT with primary antibodies (p65-specific primary antibody (1000× diluted, Cat. Number: 622602, BioLegend, San Diego, CA, USA) or β-actin-specific primary antibodies (1000× diluted, Cat. Number: 664801, BioLegend, San Diego, CA, USA)). The membrane was then incubated for 30 min at RT with appropriate secondary antibodies, before being visualized with an enhanced chemiluminescence reagent (ECL, Bio-Rad, Hercules, CA, USA).

### 4.10. NF-κB DNA Binding Assay

The NF-κB p65 transcription factor activity was determined using the NF-κB transcription factor assay kit (Colorimetric, ab207216) (Abcam, Cambridge, MA, USA) following the manufacturer’s instructions. THP-1-derived macrophages were treated for 4 h with Act d 1 (16.62 µM), LPS (0.1 µg/mL), or LPS (10 µg/mL). To investigate the inhibitory effect of small molecules, cells were treated for one hour with VA and LA (5 μM and 25 μM, respectively) prior to Act d 1 or LPS treatments. The nuclear fractions were then isolated using the manufacturer’s recommended protocol. The total protein concentration in the samples was measured using the BCA Protein Assay Kit (Thermo Fisher Scientific, Waltham, MA, USA). For the ELISA, the nuclear extracts (18 µg per well) were incubated in a plate coated with a double-stranded DNA (dsDNA) sequence containing the NF-κB response element for 1 h at RT before being incubated with a primary anti-NF-κB p65 antibody for 1 h at RT. After the addition of the HRP-conjugated secondary antibody and developing solution, absorbance was measured at 450 nm using a microplate reader (BioTek Synergy LX Multimode Reader, Agilent, CA, USA) following the addition of stop solution to each well.

### 4.11. Quantification and Statistical Analysis

For all graphs, the results are presented as mean ± SEM. Abbreviations to indicate p-values are as follows: * *p* value < 0.1, ** *p* value < 0.01, *** *p* value < 0.001, and **** *p* value < 0.0001. Statistical analyses and graphing were completed by using GraphPad Prism version 8.0.1 (GraphPad, La Jolla, CA, USA). For the analysis of statistical significance between groups, one-way analysis of variance (ANOVA) was used. Selected figures were created with Biorender, Biorender.com (accessed on 3 November 2023). All experiments, including the analysis of gene expression, microscopy, Western blot, and ELISA, were independently performed at least 3 times.

## Figures and Tables

**Table 1:** Human primer sequences used in PCR analysis.

Target Gene	Reference	Human Primer Sequence (5′-3′)	AT (°C)
*GAPDH*	NM_009386.2	For: CGAGGCATCATCCCAAATAAGAAC Rev: TCCAGAAGTCTGCCCGATCAC	65
*IL-1β*	NM_000576.2	For: AACCTCTTCGAGGCACAAGG Rev: GGCGAGCTCAGGTACTTCTG	49
*IL-25*	NM_022789.3	For: CCAGGTGGTTGCATTCTTGG Rev: TGGCTGTAGGTGTGGGTTCC	49
*TNF-α*	NM_000594.3	For: GACAAGCCTGTAGCCCATGT Rev: CTCTGATGGCACCACCAACT	49

## Data Availability

The data presented in this study are available on request from the corresponding author.
